# Plasma Saturated and Monounsaturated Fatty Acids in Behçet’s Disease

**DOI:** 10.2174/1874312901812010139

**Published:** 2018-08-31

**Authors:** Meriam Messedi, Manel Naifar, Sahar Grayaa, Faten Frikha, Mariem Messoued, Mohamed Marouene Sethom, Moncef Feki, Naziha Kaabach, Zouheir Bahloul, Kamel Jamoussi, Fatma Ayedi

**Affiliations:** 1 Unit of Research Molecular Bases of Human Diseases, 12ES17, Faculty of Medicine of Sfax, University of Sfax, 3029 Sfax, Sfax, Tunisia; 2 Biochemistry laboratory, Habib Bourguiba University Hospital, 3029 Sfax, Sfax, Tunisia; 3 Internal Medicine Department, Hedi Chaker Hospital, 3029 Sfax, Sfax, Tunisia; 4 Faculty of Medicine of Tunis, Biochemistry laboratory, La Rabta Hospital and UR05/08-08, Tunis 1007, Tunisia

**Keywords:** Behçet’s disease, Plasma saturated fatty acids, Plasma monounsaturated fatty acids,stearoyl-CoA desaturase, Inflammation, C reactive proteine

## Abstract

**Background:**

Fatty Acid (FA) composition of serum has been associated with many markers of inflammation. In this study, we tried to examine plasma Saturated Fatty Acid (SFA) and Monounsaturated Fatty Acid (MUFA) composition in Behçet's Disease (BD) patients. The associations between the circulating FA levels and some markers of inflammation have also been investigated.

**Methods:**

This study is a cross-sectional one. In fact, a total of 101 BD patients and healthy controls group of 99 subjects are enrolled. Gas Chromatograph equipped with a Capillary Split/Splitless Injector and flame ionization detector was used to analyze the plasma SFA and MUFA compositions. The high sensitivity C-Reactive Protein (hsCRP) and fibrinogen levels were measured using standard techniques.

**Results:**

BD patients had significantly higher proportions of Mystiric Acid (MA), Palmitic Acid (PAM), Palmitoleic Acid (POA) and Stearoyl-CoA Desaturase (SCD)-16, compared to controls.

The results revealed that patients with severe involvements had high levels of POA and total MUFA associated with higher SCD-16 activity compared to those with minor ones. The receiver operator characteristic curve analysis revealed that POA could well discriminate BD patients with severe clinical manifestations. In the bivariate analysis, hsCRP was found to be positively correlated with total SAFA and POA elongase activity index but negatively correlated with SCD-18 activity index. The STA, POA, elongase and SCD-16 activity index are correlated with fibrinogen. On the other hand, the multivariate analysis showed that POA remained associated with higher levels of hsCRP.

**Conclusion:**

Unfavourable plasma SFA and MUFA profile were reported in BD patients. POA, which is associated with higher plasma hsCRP level, may play a role in the pathogenesis of BD.

## 1 INTRODUCTION

Behcet's Disease (BD) is a relapsing chronic, multi-systemic inflammatory disorder which is classified as vasculitis [1] which is mainly characterized by oro-genital ulcers and cutaneous manifestations. Ocular inflammation, articular manifestations, vascular and neurological involvements may also occur in patients with BD [1]. The pathophysiology of this disease remains unknown and many hypotheses are intended to elucidate its etiology. Recent data suggest that BD is thought to link genetic, immuno-metabolic, inflammation and infection factors [1, 2]. In fact, BD is characterized by markedly high levels of inflammatory markers including C-Reactive Protein (CRP), TNF-α and IL-6 among others [3-5] and of activated inflammatory cells at the site of tissue damage and in the systemic circulation [1, 6].

Actually, several studies support the idea that a variety of dietary factors, which can modulate inflammation, are associated with specific biomarkers of inflammation, which promote the deregulation of specific immune cell subpopulations [7]. Among the many fat species, Fatty Acids (FA) seem to be the smallest but play an essential role in many cell functions. In this context, numerous studies have reported that dietary and/or blood FA compositions are associated with systemic inflammation, oxidative stress and impaired endothelial function [7, 8]. FA are energy sources and major components of cell membrane that can influence human health and well-being as well as disease risk. Moreover, they are involved in the regulation of membrane function and structure; intracellular signaling pathways, gene expression and the bioactive lipid mediators production [7]. In fact, some effects of FA on inflammatory process seem to be mediated by, or at least are associated with, modifications in fatty acid composition of plasma and cell membranes. FA belong to a chemically heterogeneous group and are classified according to their degree of desaturation, *i.e.*, Saturated FA (SFA), Monounsaturated FA (MUFA), Polyunsaturated FA (PUFA);. However, the issue of causality and the degree to which each FA might contribute and serve to promote inflammation remain fully unsolved. The pro or anti-inflammatory effects of FA depend on their chemical structure (chain long, the number and the position of double bounds). On the other hand, several observational studies have reported that n-6 PUFA tend to have a pro-inflammatory role, as they are the precursors of active pro-inflammatory molecules such as prostaglandins or leukotrienes [9], whereas, n-3 PUFA have been found to diminish systemic inflammation [10]. These FA can generate resolving lipids which enhance resolution mechanisms in inflammation [9]. As for MUFAs, they are widely believed to be beneficial to health, by favorably altering serum lipids and inflammation [11, 12]. However, data from cell culture have suggested that MUFA have deleterious effects on the inflammatory pathways [13, 14]. Regarding the SFA, most of the clinical trials, cohort and cell culture studies corroborate of their pro-inflammatory effects [15-17]. Furthermore, it has been demonstrated that SFA are associated with a high risk of many chronic inflammatory diseases such as diabetes [18], metabolic syndrome [19] and atherosclerosis [20]. Indeed, the collected data showed that plasma lipid SFA level is positively correlated the plasma levels of the CRP, fibrinogen and pro-inflammatory cytokines [21, 22]. In fact, SFA seem to stimulate inflammatory responses through immune cell effector activation and recruitment [23, 24].

Considering that inflammatory process and immune cells; which play a pivotal role in the physiopathology of BD; are sensitive to diet tone changes, we intended to examine plasma saturated and monounsaturated FA compositions in Tunisian BD patients. The associations between individual FA and some markers of inflammation were also performed. As such, investigations of these patients would generate new information regarding the relationships between individual FA and inflammation prior to the onset of this chronic inflammatory condition.

## 2 PATIENTS AND METHODS

### 2.1 Population Study

Tunisian BD patients, who fulfilled the criteria of the International Study Group Criteria of BD, were enrolled [25]. Samples from gender, age and Body Mass Index (BMI)-matched group of healthy controls were recruited. Clinical data about the onset of the disease, medication history, and clinical manifestations were ascertained. The clinical evaluation included information about smoking history, alcohol consumption, familial history of premature cardiovascular disease, diabetes mellitus, hypertension, and any use of medication. The BD’s current activity form was determined for each patient at the time of the blood withdrawal. The BD Activity Index (BDAI) was calculated as previously reported [26]. Severe BD was defined based on clinical manifestation. The patients eligible for this group were whose had serious vital organ involvement including ocular damages, neurological lesions, vascular and gastrointestinal involvements [27, 28]. This research study has been approved by the local ethics committee and all the participants provided informed written consent before being enrolled in this study.

### 2.2 Analytical Methods

#### 2.2.1 Food Intake Assessment

Detailed food consumption was assessed using a validated semi quantitative food frequency questionnaire adapted to the Tunisian context [29]. These data were collected through a face-to-face interview with all the participants. The Tunisian food composition database has been used to convert dietary intake data into nutrient values [30]. Then, all the calculations were performed using Bilnut software version 2.01 (S.C.D.A. Nutrisoft, Cerelles, France). The dietician registered all the meals and beverages consumed in one week for a period of one month. For the estimation of the size of individual portions, a picture booklet and household measurement units were provided to each participant. In order to determine the frequency consumption of the different food intake, participants were asked about the proportion of each item consumed per day.

#### 2.2.2 Blood Processing and Fatty Acid Analysis

After an overnight fasting, whole blood specimens were collected into sodium citrate, heparin and EDTA tubes. Plasma from heparin-containing tubes was immediately used for glycaemia, lipid and high-sensitivity C-Reactive Protein (hsCRP) analyses. Glycaemia was measured using the glucose oxidase method [31]. Serum levels of total cholesterol, triglycerides and high-density lipoprotein cholesterol were measured by standard enzymatic methods. Analyses were carried out on ARCHITECT c8000 analyzer (Abott Diagnostics, Abbott Park Rd, US) using corresponding reagent kits. The low-density lipoprotein cholesterol was calculated using the formula of Friedewald [32]. Apolipoprotein A-I andapolipoprotein B 100 levels were measured by turbidmetric immunoassay using COBAS INTEGRA 400 analyzer (Roche Diagnostics Basel, Switzerland). Plasma hsCRP concentrations were measured with a particle-enhanced immune-turbidimetric assay (Roche Tina-Quant CRP, Roche Diagnostics, Basel, Switzerland). Samples of citrate plasma were analyzed within 2 h of venipuncture by an automatic coagulometer (Multifibren, BCS, Siemens Healthcare Diagnostics) for fibrinogen measurements.

#### 2.2.3 Analysis of Plasma FA Using Gas Chromatography

For FA, plasma from EDTA-tubes was used. In fact, plasma samples were separated within 1 hour by centrifugation at 1000×*g* for 10 min, followed by the addition of an antioxidant butylated hydroxyl toluene and stored at -80°C. FA were analyzed using a gas chromatography model 6890N (Agilent Technologies), equipped with a split/splitless capillary Intel system and a flame ionization detector. A polyethylene glycol fused silica capillary column (Innowax, 30 m 9 0.25 mm 9 0.25 lm film thickness) purchased from Agilent (Wilmington, Delaware, USA) was used. The injector and detector temperatures were 230 and 280°C, respectively. The flow-rate of nitrogen as the carrier gas was set to 1.5 ml/min in a constant flow mode. The oven temperature was held at 150°C for 1 min, then programmed at a rate of 15°C/min to increase up to 210°C and kept constant during 5 min then subsequently programmed at 250°C with a rate of 4°C/min [33].

Peak areas were quantified using chromatography software (Agilent Technologies Chem Station FamilyR data analysis). The fatty acid methyl esters, which were first identified by comparing their retention times to those of known standards and results, were expressed as relative percentages of each FA.

As estimated enzyme activities from serum fatty acid ratios has been shown to approximate liver enzyme activities [34], elongase and desaturase indices were estimated according to the following:elongase=C18:0/C16:0; Stearoyl-CoA Desaturase-18 (SCD-18)=C18:1 n-9/C18:0, Stearoyl-CoA Desaturase-16 (SCD 16)= C16:1n-7/C16:0.

In the current study, the authors examined the plasma SFA and MUFA levels using relative percentage (% of total FA) and absolute concentration (µg/ml) methods. The long-term imprecision (CV) was less than 10% for each fatty acid [33].

### 2.3 Statistical Analysis

Statistical analyses were performed using the SPSS software package (version 20.0 for windows; SPSS Inc. Chicago, IL, USA). Continuous variables were given as the mean (standard deviation) and analyzed by Student *t*-test. Since our cohort showed a non-Gaussian distribution, some non-parametric statistical methods (Mann–Whitney *U*- and chi-squared tests) were used to analyze the data and results were expressed as median (25th–75th percentile). Receiver Operating Characteristic Curves (ROC) was performed to identify a predictive markers for diagnostic of severe form of BD. Area Under the Curve (AUC), cutoff values together with degree of specificity and sensitivity were calculated. Pearson's and Spearman's correlation tests were used to evaluate the associations between continuous variables with Gaussian distribution and non-Gaussian distribution, respectively. The regression models adjusted for age, gender, BMI, smoking and lipid parameter levels were used to evaluate the associations between plasma SFA and MUFA and the markers of inflammation. The odds ratios and 95% Confidence Intervals (CIs) for FA were calculated by binary logistic regression models. The reported *p-*values were based on two-tailed calculations and values less than 0.05 were considered as a statistically significant level.

## 3 RESULTS

### 3.1 Studied Population Characteristics

A cohort of 142 BD patients and 139 healthy controls were enrolled. All the subjects with a history of early cardiovascular disease, hypertension, diabetes, dyslipidaemia, overweight, smoking, drinking and any other chronic inflammatory condition as well as any subjects treated with Statins, asprin or corticoids; at least one month before the blood withdrawal, have been excluded. After a selection operation, the studied population consists of 101 BD patients (72 men and 29 women) and 99 healthy controls (70 men and 29 women). The clinical features of BD patients were summarized in Table **1**. Anthropometric, demographic and clinical variability including sex ratio, age, systolic and diastolic blood pressures, BMI measurement were similar in both groups. The laboratory values did not differ between the BD patients and the controls except for plasma high-density lipoprotein cholesterol level (1.23mmol/L vs 1.11mmol/L, *p*=0.02) and hsCRP (2.6 mg/mL vs 1.37 mg/mL, *p*=0.001) (Data not shown).

### 3.2 Fatty Acid Profile

**Fig. (1):**
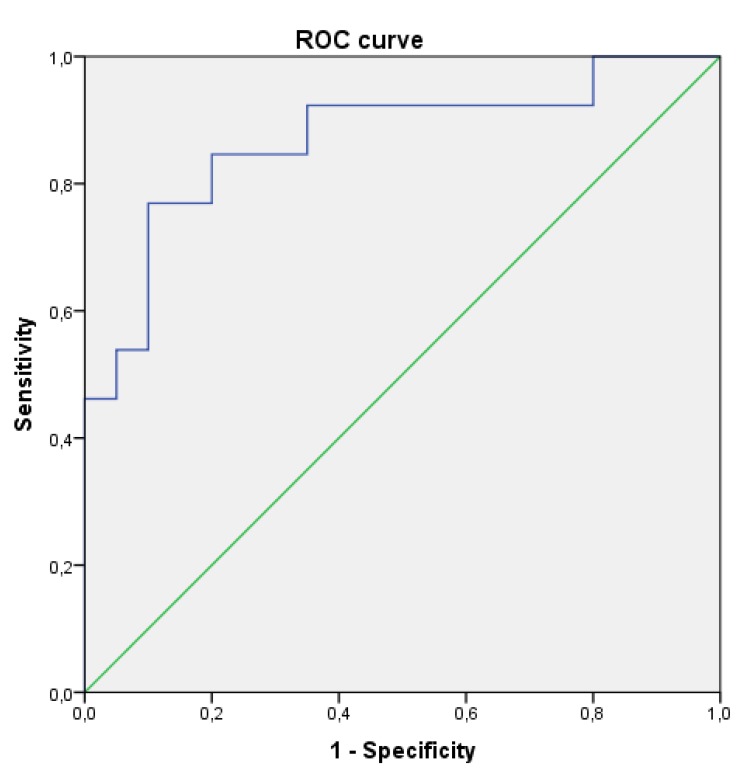
Receiver Operating Characteristic (ROC) curve analysis of plasmatic palmitoleic acid to predict severe form of Behcet’s Disease.

Actually, no significant difference was observed between the two studied groups regarding the dietary intake, energy intake, macro- and micro- nutriments, and dietary FA (Table **2**). On the other hand, plasma fatty acids composition was significantly different between the BD patients and the healthy controls. In fact, BD patients had significantly higher proportions of Mystiric Acid (MA), Palmitic Acid (PAM) and total SFA compared to the controls group. Moreover, no difference was observed regarding Stearic Acid (STA) or Oleic Acid (OLA). The expected SCD-16 activity was markedly higher in patients compared to the controls but no significant difference was detected for the other studied enzymatic activity indices (Table **3**).MUFA and Palmotoleic Acid (POA) were significantly more abundant in BD patients (Table **2**). BD patients with severe form had also higher concentrations of POA and total MUFA associated with increased SCD-16 activity (Table **4**). A Receiver Operator Characteristic (ROC) analysis, corresponding to POA concentrations in plasma as biomarker of severe form, showed an area under curve of 0.85 (95% confidence interval: 0.72–0.99, *p*-value = 10^-3^) and a predictive threshold value of 108 µg/mL. The found sensitivity and specificity of POA found were 92% and 65%, respectively (Fig. **1**).

### 3.3 Correlations between Plasma Fatty Acids and Inflammation Markers in BD Patients

In BD patients group, hsCRP was positively correlated with elongase activity index (absolute value), total SAFA and POA (relative values) but negatively with SCD-18 activity (absolute value). The MA, PAM and STA were not associated with hsCRP when expressed as relative and absolute values (Table **5**), while the STA; in its relative and absolute values; was only correlated with fibrinogen.

On the other hand, the POA, elongase and SCD-16 activities were also correlated also with fibrinogen when expressed as absolute and relative values. However, a negative correlation was found between the relative value of SCD-18 and fibrinogen (Table **5**). Moreover, the multiple linear regression adjusted for age, gender, BMI, smoking and lipid parameter levels revealed that the POA remained significantly associated with higher hsCRP (Table **6**). However, no association with fibrinogen has been reported.

## 4 DISCUSSION

In the current study, we have examined circulating SFA and MUFA levels and laboratory data. It is worth noting that in our study, we have used plasma FA to assess their concentrations in the human body. Because FA acids in the blood compartments and tissues are highly correlated [35], plasma FA levels should reflect their concentrations in the tissues. It is well known that differences in diets would influence the blood and tissue composition of some fatty acids. Therefore, the dietary intake in the two studied population was assessed. Since the dietary intake was similar, our results of altered FA profile may be explained, at least in part with the metabolic disturbances of the BD patients.

BD: Behcet Disease; MA: Mystiric. PAM: Palmitic Acid; STA: Stearic Acid; POA: Palmitoleic Acid; OLA: Oleic Acid; SFA: Saturated Fatty Acids; MUFA: Monounsaturated Fatty Acids; SCD: Stearoyl CoA Desaturase; %: percentage; µg:microgram ; ml: milliliter;µmol: micromole; *: p ≤ 0,05; **: *p* ≤ 0,001.

MA: mystiric. PAM: palmitic acid; STA: stearic acid; POA: palmitoleic acid; OLA: oleic acid; SFA: saturated fatty acids; MUFA: Monounsaturated fatty acids; SCD: stearoyl CoA desaturase; hsCRP: high-sensitivity C-reactive protein; % percentage; µg: micrograms; ml: milliliter; *: *p* ≤ 0.05; ** *p* ≤ 0.001.

Moreover, higher MA, PAM and total SFA levels were observed in BD compared to the healthy control subjects. In fact, BD is a systemic inflammatory disorder characterized by immunological abnormalities [36]. The immune system effectors cells, mainly neutrophils and macrophages sense nutrient imbalances. SFA are believed to substantially promote to pro-inflammatory state. Besides, the SFA could exacerbate inflammatory responses through various mechanisms. On the other hand, in vitro studies, have reported that SFA, mainly PA, activate inflammatory signaling, including the Nuclear Factor κB (NF-κB) pathway and enhance the inflammatory cytokines synthesis in the vasculature and in leukocytes [37-39]. Moreover, SFA may modulate cytokines production through epigenetic modifications of their DNA promoter [40]. Additionally, SFA were shown to incapable of increasing Nitrous Oxide (NO) production and cyclooxygenase-2 (COX-2) mRNA expressions [41, 42]. High levels of SFA, which were reported in our BD patients, may enhance the pro-inflammatory condition and chronic inflammation, hallmarks of BD [43]. This seems to be in conformity with the data suggesting that SFA are associated with chronic inflammatory disease such as Crohn’s disease [44] and psoriasis [45].

Our results also reported increased level of POA, a view which was supported by enhanced SCD-16 activity also observed in BD patients. Severe form of BD was associated with higher levels of POA, total MUFA and SCD-16 activity. Interestingly, a high plasma level of POA also seems to be a good predictor of severe form of BD. Furthermore, POA and SCD-16 were positively correlated with the high level of hsCRP while the proportion of POA remained strongly associated with this marker even after multivariable adjustment.

In the human body, the dietary origin of POA is negligible because of its rapid oxidation after absorption. The tissue and blood concentrations of POA mainly reflect de *novo* lipogenesis mediated by SCD, a key enzyme of the biosynthesis of MUFA from SFA. The POA and SCD, have been recently gaining increasing attention. It has been shown that high levels of POA and SCD-16 activity were associated with many inflammatory conditions such as Crohn’s [44] and chronic kidney [46, 47] diseases, cancers [48, 49] and diabetes [50]. The biological mechanisms through which POA or SCD-16 activity might exert their effects on inflammation are still unclear. Probably, the most interesting evidence is generated from the longitudinal study of Petersson et *al*. [51], in which POA as well as SCD-1 at the age of 50 are associated with CRP measured 20 years later in a cohort of 767 men. Such an association has been observed in Finnish 1373 men [52].These results are in line with the findings of Stryjecki [53] who revealed a positive association between POA and CRP levels in females of both European and Asian descendants. Similar to our results, the reported associations in these studies remained significant after adjusting for covariates (*e.g.* age, BMI, gender, energy intake), arguing that POA is independently associated with an inflammatory state. It is not fully understood why SCD-16 and its product, POA, are associated with CRP. Nevertheless, the regulation of inflammatory pathways is extremely complex and occurs as a result of both cellular crosstalk and intracellular signaling pathways. Previous findings have demonstrated that POA up-regulate both the expression and synthesis of pro-inflammatory cytokines mainly Interleukin-6 (IL-6) and tumor necrosis factor- α (TNF-α) [54, 55] *via* NFκB activation [56, 57]. Moreover, the experimental data revealed that pro-inflammatory cytokines (IL-1, IL-6, TNF-α) synergistically acting would induce CRP gene transcription by controlling nuclear transcription factors [58, 59].

It is also possible that inflammation could be enhanced by a metabolic disorder associated with elevated POA level and SCD-1 activity. Indeed, several human observational studies showed that high POA concentrations in various tissues are linked to unfavorable metabolic outcomes. According to the Uppsala Longitudinal Studies, POA in adipose tissue and in serum was associated with an increased risk of developing metabolic syndrome after 20 years of follow-up in 706 men [47]. Furthermore, POA was correlated with several cardiometabolic risk factors, including higher BMI and blood pressure, plasma total cholesterol, triglycerides, apolipoprotein A-I, apolipoprotein Band endothelial dysfunction [60, 61]. The existing data suggest that the products of SCD-1 (OLA and POA), are essential components for the production of triglycerides and cholesterol esters [62]. As a consequence, this might clarify the association of POA with increased triglycerides and cholesterols levels in human studies [62].

On the other hand, several studies have demonstrated the beneficial role of POA in inflammation and metabolic outcomes. For example, healthy participants who received a 30days supplementation of purified POA, showed reduction of CRP, triglyceride and of low-density lipoprotein levels and a significant increase in high-density lipoprotein concentration [63]. In another study conducted on 20 patients with ulcerative colitis, an eight week treatment of Cis-POA seems to decrease inflammation through the decrease of hsCRP level [64].

On the other hand studies conducted on animal showed favorable effects of POA. The mice macrophages treated with POA showed a decreased of NF-κB phosphorylation and reduced expressions of pro-inflammatory cytokines [65-67]. It has been also shown that POA contribute to decrease the levels of pro-inflammatory cytokines (IL-1β, IL-6 and TNF-α) [55, 68] as well as the expression of TLR4 [68] in obese mice liver.

The contradicting findings of the POA effect on the inflammatory process and metabolic disturbance from available data deserve to be discussed. One explanation may be due to the different forms of POA (Cis or Trans, un- or esterifed) examined in the studies. The POA as a free fatty acid is used or measured in animal and human in vitro studies showed positive effects [64, 66]. However, when measured in esterified form, the POA may not show its anti-inflammatory effects [52]. Furthermore, studies have shown that lipogenesis is quite different in men and mice models [69]. Therefore, more well-designed studies are required to elucidate on the mechanisms underlying the potentially various associations of POA-in different tissues or in different forms-with inflammation and metabolic abnormalities.

In fact, there are several points to be considered in the present study. Firstly, the observational cross-sectional design did not help identify any causative relationship. Another obvious limitation is the use of fibrinogen and CRP as markers of inflammation. The latter are commonly used because their concentration in plasma is relatively elevated and well above the lowest limit of detection. However, they are considered as non-specific indicators of inflammation and consequently give no information concerning the origin of the inflammatory process. Future research may consider incorporating additional specific markers of inflammation such as IL-1, IL-6 and TNF-α.

## CONCLUSION

BD is a chronic, multi-systemic inflammatory disorder with uncertain pathogenesis. Our objective in this study is to investigate the plasma saturated and monounsaturated FA in BD and its relationship with some inflammatory markers. Data showed that an altered FA profile and enzymes activities would enhance inflammation which would provide important insights into the FA metabolism in BD as well as guide for future biomarker selection. Further researches in larger BD populations in other ethnics with different dietary intake are required to confirm our findings. We would suggest that if our findings are confirmed in future studies, pharmacological interventions targeting SCD-1 activity and level of POA could be considered.

## Figures and Tables

**Table 1:** Clinical features of BD patients.

Characteristics	n=101
Disease duration, months	124 (2 –326)
Genital ulcer, n (%)	80 (80%)
Papulopustular lesions, n (%)	82 (81%)
Erythema nodosum-like lesions, n (%)	10 (9%)
Positive pathergy test, n (%)	48 (47%)
Arthritis, n (%)	38 (37%)
Ocular involvement, n (%)	39 (38%)
Posterior uveitis, n (%)	20 (19%)
Panuveitis, n (%)	16 (15%)
Retinal vasculitis, n (%)	24 (23%)
Deep vein thrombosis, n (%)	26 (25%)
Neurological involvement, n (%) Gastrointestinal involvement, n (%)	23 (22%) 3 (3%)

%: percentage, n: number.

**Table 2:** Dietary intake comparison in the studied populations.

**Parameters**		**Healthy Controls (n=99)**		**BD Patients (n=101)**		***P-* Value**	
**Energy** Total energy (Kcal)		3265 (2375.75-4300.50)		3297 (2452.5-4300)		0.75	
**Macronutrients**							
Protein (g/day)		98.8 (10.2)		91.2 (9.1)		0.33	
Carbohydrates (g/day)		392.1 (276.9-432.7)		396.9 (301.3-444.1)		0.18	
Fiber (g/day)		27.26 (19.45-38.9)		24.3 (15.87-39.2)		0.26	
Total fat (g/day)		88 (71.8-102.3)		94 (77.56-120.3)		0.36	
Total SFA (%)		11.45 (9.77-13.05)		10.38 (8.96-12.26)		0.21	
Total MUFA (%)		14.81 (11.49-17.74)		14(9.71-19.59)		0.19	
Total PUFA (%)		11.77 (4.93-18.23)		12.44 (5.01-20.02)		0.33	
**Micronutrients (/day)**							
Calcium (mg)		542 (301.5-789)		578.5 (437.5-826)		0. 54	
Iron (mg)		14.18 (11.28-17.05)		13.42 (10.12-17.1)		0.31	
Vitamin B12 (µg)		5.4 (4.5-6.7)		4.5 (3.9-6.4)		0.57	
Vitamin C (mg)		98 (59.5-171.5)		89 (45-150)		0.5	
Vitamin E (mg)		13.7 (8.44-30.5)		14.23 (10.56-31.20)		0.59	
Folates (µg)		223.4 (191.25-271.15)		225.8 (205.25-274.72)		0.8	
Sodium (mg)		2692 (2463.25-3206.75)		2536 (2315-2939.5)		0.21	
Potassium (mg)		2963 (2624-3683.25)		2827 (1588-2306.5)		0.42	
Phosphore (mg)		1210.5 (1026.25-1475)		1116 (957.5-1389.75)		0.49	
Magnesium (mg)		296 (204.25-347)		307 (223-448)		0.4	
Zinc (mg)		9.29 (8.48-10.96)		9.52 (8.88-11.59)		0.31	

SFA: Saturated Fatty Acids; MUFA: Monounsaturated Fatty Acids; PUFA: Polyunsaturated Fatty Acids; Kcal: Kilocalories; g:gram; %: percentage; mg: milligrams; µg: micrograms; /: per.

**Table 3:** Plasma saturated and monounsaturated fatty acids profile and estimated enzymatic activities indices in BD patients and controls.

-	**Controls (n=99)**		**BD Patients (n=101)**	
-	**%**	**µg/ml**	**%**	**µg/ml**
MA (C14:0)	**0.63 (0.50-0.84)**	**26.28 (17.87-40.28)**	0.75 (0.55-0.98)*	27.96 (19.46-42.49)*
PAM (C16:0)	**21.87 (20.25-23.26)**	**856.17 (622.15-1082.05)**	22.31 (20.87-24.88)*	881.54 (664.0-106.69)*
STA (C18:0)	6.09 (5.26-6.70)	237.45 (182.05-304.20)	6.04 (5.31-7.59)	233.11 (174.75-311.84)
Total SFA	**28.49 (26.67-30.98)**	**1070.70 (891.52-1376.28)**	29.65 (27.24-32.43)*	1156.77 (818.69-1408.79)*
POA (C16:1n-7)	**2.94 (2.01-4.26)**	**107.27 (72.56-168.15)**	3.88 (2.72-5. 51)**	130.13 (89.08-188.76)*
OLA (C18:1n-9)	21.5 (19.79-23.66)	639.81 (465.18-892.30)	20.7 (18.78-23.28)	584.72 (429.67-827.62)
Total MUFA	24.73 (22.88-27.09)	707.27 (543.81-976.93)	25.25 (22.52-28.4)	741.23 (536.35-1064.83)
Elongase	0.28 (0.24-0.31)	0.28 (0.25-0.31)	0.27 (0.23-0.32)	0.27 (0.23-0.32)
SCD-16	**0.13 (0.09-0.19)**	**0.12 (0.09-0.178)**	0.17 (0.12-0.24)**	0.15 (0.11-0.21)*
SCD-18	3.55 (2.91-4.49)	2.73 (2.27-3.46)	3.30 (2.53-4.37)	2.53 (1.98-3.40)

BD: Behcet Disease; MA: Mystiric. PAM: Palmitic acid; STA: Stearic acid; POA: Palmitoleic acid; OLA: Oleic acid; SFA: Saturated fatty acids; MUFA: Monounsaturated fatty acids; SCD: Stearoyl CoA desaturase; %: percentage; µg:microgram ; ml: milliliter;µmol: micromole; *: p ≤ 0,05; **: *p* ≤ 0,001.

**Table 4:** Comparison of plasma saturated and monounsaturated fatty acids profile and estimated enzymatic activities indices between BD patients with minor and with severe clinical manifestations.

-	**BD patients with minor clinical manifestations (n=57)**		**BD patients with severe clinical manifestations (n=44)**	
-	**%**	**µg/ml**	**%**	**µg/ml**
MA (C14:0)	0.60 (0.45-0.85)	26.09 (15.86-32.72)	0.80 (0.59-0.94)	29.24 (21.87-45.71)
PAM (C16:0)	22.31 (20.74-24.59)	720.26 (647.02-952.61)	21.78(20.56-24.62)	916.79(732.59-1092.74)
STA (C18:0)	6.11(5.32-7.62)	213.28(156.72-276.56)	5.92(4.86-7.27)	227.77(174.78-329.20)
Total SFA	29.42(27.14-33.22)	959.79(832.17-1236.87)	28.23 (25.98-31.76)	1152.89(1011.23-1461.96)
POA (C16:1n-7)	**3.56 (2.18-5.02)**	**101.33 (76.60-144.29)**	5.11 (3.61-6.33)**	191.64 (159.49-252.41)**
OLA (C18:1n-9)	20.15 (18.57-22.70)	542.40 (400.92-672.51)	22.0(20.59-27.20)	699.96 (570.11-1043.40)
Total MUFA	**23.81 (21.34-27.13)**	**638.50(512.01-787.02)**	27.69 (25.45 -30.62)*	941.61(734.08-1265.42)**
Elongase	0.27 (0.24-0.31)	0.28 (0.25-0.32)	0.27 (0.22-0.32)	0.25 (0.21-0.31)
SCD-16	**0.15 (0.09-0.21)**	**0.13 (0.08-0.19)**	0.23(0.15-0.30)**	0.21 (0.15-0.27)**
SCD-18	3.17 (2.56-4.28)	2.48 (2.05-3.28)	3.81(3.11-5.06)	3.43 (2.61-3.88)

**Table 5:** Correlations between plasma saturated and monounsaturated fatty acid s and studied inflammation markers in Behcet’s disease.

**Plamsa FA**	**hsCRP**		**Fibrinogen**	
	**Absolute** **(µg/ml)**	**Relative** **(%)**	**Absolute (µg/ml)**	**Relative** **(%)**
MA (C14:0)	-0.111	-0.082	-0.036	-0.052
PAM (C16:0)	0.062	0.147	0.181	0.131
STA (C18:0)	0.185	0.09	0.302**	0.408**
Total SFA	0.188	0.226*	0.110	0.159
POA (C16:1n-7)	0.178	0.261*	0.302*	0.271*
OLA (C18:1n-9)	-0.154	-0.081	-0.038	-0.050
Total MUFA	-0.157	-0.065	-0.083	-0.025
Elongase	0.276*	0.127	0.380**	0.313**
SCD-16	0.183	0.185	0.217*	0.254*
SCD-18	-0.264*	-0.053	-0.174	-0.248*

**Table 6:** Logistic regression that evaluate the association between palmitoleic acid andhsCRP.

**Independent**	**hsCRP^a^**	
-	**Model 1: β (95% CI)**	**Model2: β (95% CI)**
POA (C16:1n-7)	31.08 [2.41-400.59]	11.42 [1.25-85.64]

Logistic regression (Wald) was used for multivariate analysis, adjustment co-variables are age, gender, BMI, smoking and lipid parameter levels. POA: Palmitoleic Acid, hsCRP: high-sensitivity C-Reactive Protein; ^a^hsCRP was modeled as categorical variable stratifying hsCRP levels into two groups according to the mean value observed (*i.e.*, 2.6); model 1 when variables are expressed as absolute values, *p*-value= <10^-3^ ; model 2 when variables are expressed as relative values, *p*-value= <10^-3^ ;CI: Confidence Interval
